# Intersectionality of Sexual Orientation, Race, and Ethnicity in Medical School Attrition

**DOI:** 10.1001/jamanetworkopen.2025.14515

**Published:** 2025-06-10

**Authors:** Mytien Nguyen, Dowin Boatright, John Paul Sánchez, Alexandra M. Hajduk, Shruthi Venkataraman, Meghan O’Connell, Allison Aviles, Pradeep Rajbhandari, Sarwat I. Chaudhry

**Affiliations:** 1 Department of Immunobiology, Yale School of Medicine, New Haven, Connecticut; 2 Department of Emergency Medicine, New York University Grossman School of Medicine, New York; 3 Universidad Central Del Caribe School of Medicine, Bayamón, Puerto Rico; 4 Section of Geriatrics, Department of Internal Medicine, Yale School of Medicine, New Haven, Connecticut

## Abstract

This cross-sectional study examines the association of the intersectionality of sexual orientation, race, ethnicity, and sex with attrition rates of students from medical school using national cohort data.

## Introduction

Disparities in medical school attrition undermine efforts to develop a diverse physician workforce. While Black, Hispanic, Indigenous, and low-income students may be more likely than their non-Hispanic White peers to leave medical training,^1^ little is known about attrition rates for students identifying as lesbian, gay, and bisexual (LGB). We examined attrition rates at the intersection of sexual orientation, race, ethnicity, and sex among a national cohort of medical students.

## Methods

This cross-sectional study used 2014 to 2017 data from the Association of American Medical Colleges data warehouse. As data were deidentified, the Yale University Institutional Review Board deemed the study exempt from review and informed consent. The study followed the STROBE reporting guideline.

We defined attrition as dismissal or withdrawal from medical school for any reason reported in student records. Sexual orientation and age were self-selected on the Matriculating Student Questionnaire (MSQ) administered to first-year students. Students self-reported age, sex, race, and ethnicity on their medical college application. Matriculants took both the 1991 and 2015 versions of the Medical College Admission Test (MCAT). Quartiles were computed for each MCAT version, and a final quartile variable was created by combining the 1991-2015 quartiles to minimize bias.

We used multivariable logistic regression to estimate the odds of attrition by LGB identity and intersectional LGB, sex, racial, and ethnic identities, adjusting for age, MCAT, and grade point average quartiles. A 2-sided *P* < .05 defined statistical significance. Analyses were performed between December 2024 and January 2025 using Stata, version 18.0 (StataCorp).

## Results

Among 83 342 matriculants, 56 031 (67.2%) responded to the MSQ, and attrition rates were higher among MSQ nonrespondents (3.8% vs 2.6%; *P* < .001). Among respondents, 45 296 (80.8%) with complete demographic data were included in the analysis (35.6% aged ≤22 years and 64.3% aged ≥23 years; 51.0% female and 49.0% male; 19.0% identifying as Asian, 6.2% as Black, 6.2% as Hispanic, 57.9% as White, and 10.5% as other race and ethnicity; 6.3% identified as LGB) (Table). Overall, 1147 students (2.5%) left school. Compared with non-LGB students, a higher proportion of bisexual (4.2% vs 2.4%; adjusted odds ratio [AOR], 1.99 [95% CI, 1.51-2.62]; *P* < .001) and gay and lesbian (3.7% vs 2.4%; AOR, 1.47 [95% CI, 1.11-1.94]; *P* = .007) students left school.

**Table.** Association of Sexual Orientation and Race and Ethnicity With Attrition From Medical School

Characteristic	Overall, No. (%)	Attrition, No. (%)	AOR (95% CI)
No. of students (%)	45 296 (100)	1147 (2.5)	NA
Sexual orientation			
Non-LGB	42 415 (93.6)	1033 (2.4)	1 [Reference]
Bisexual	1370 (3.0)	58 (4.2)	1.99 (1.51-2.62)
Gay or lesbian	1511 (3.3)	56 (3.7)	1.47 (1.11-1.94)
Race and ethnicity			
Asian	8625 (19.0)	198 (2.2)	1.26 (1.07-1.50)
Black	2828 (6.2)	135 (4.7)	1.42 (1.16-1.75)
Hispanic	2816 (6.2)	133 (4.7)	1.53 (1.25-1.87)
White	26 249 (57.9)	544 (2.0)	1 [Reference]
Other^a^	4778 (10.5)	137 (2.8)	1.21 (1.00-1.47)
Sex			
Female	23 111 (51.0)	516 (2.2)	0.69 (0.61-0.78)
Male	22 185 (49.0)	631 (2.8)	1 [Reference]
Age at matriculation, y			
≤22	16 158 (35.6)	368 (2.2)	1 [Reference]
≥23	29 138 (64.3)	779 (2.6)	0.91 (0.80-1.04)
MCAT quartile			
First (lowest)	12 158 (26.8)	548 (4.5)	2.80 (2.30-3.40)
Second	13 620 (30.1)	291 (2.1)	1.39 (1.14-1.71)
Third	9508 (21.0)	159 (1.6)	1.10 (0.87-1.38)
Fourth (highest)	10 010 (22.1)	149 (1.4)	1 [Reference]
GPA quartile			
First (lowest)	11 646 (25.7)	441 (3.7)	1.73 (1.43-2.10)
Second	11 382 (25.1)	303 (2.6)	1.40 (1.15-1.69)
Third	11 182 (24.7)	213 (1.9)	1.05 (0.86-1.28)
Fourth (highest)	11 086 (24.5)	190 (1.7)	1 [Reference]

Abbreviations: AOR, adjusted odds ratio; GPA, grade point average; LGB, lesbian, gay, bisexual; MCAT, Medical College Admission Test; NA, not applicable.

^a^ Other race and ethnicity included American Indian, Alaska Native, Native Hawaiian, Pacific Islander, multiracial, multiethnic, and other.

In intersectional analyses, after adjusting for MCAT performance, non-LGB Asian, Black, and Hispanic male; LGB White female; and LGB Hispanic male and female students compared with non-LGB White students were more likely to leave school (Figure). Among these groups, LGB Hispanic male and female students had the highest odds of attrition (AOR, 3.11 [95% CI, 1.47-6.56]; *P* < .001; and 3.52 [95% CI, 2.02-6.15]; *P* = .003], respectively).

**Figure.**
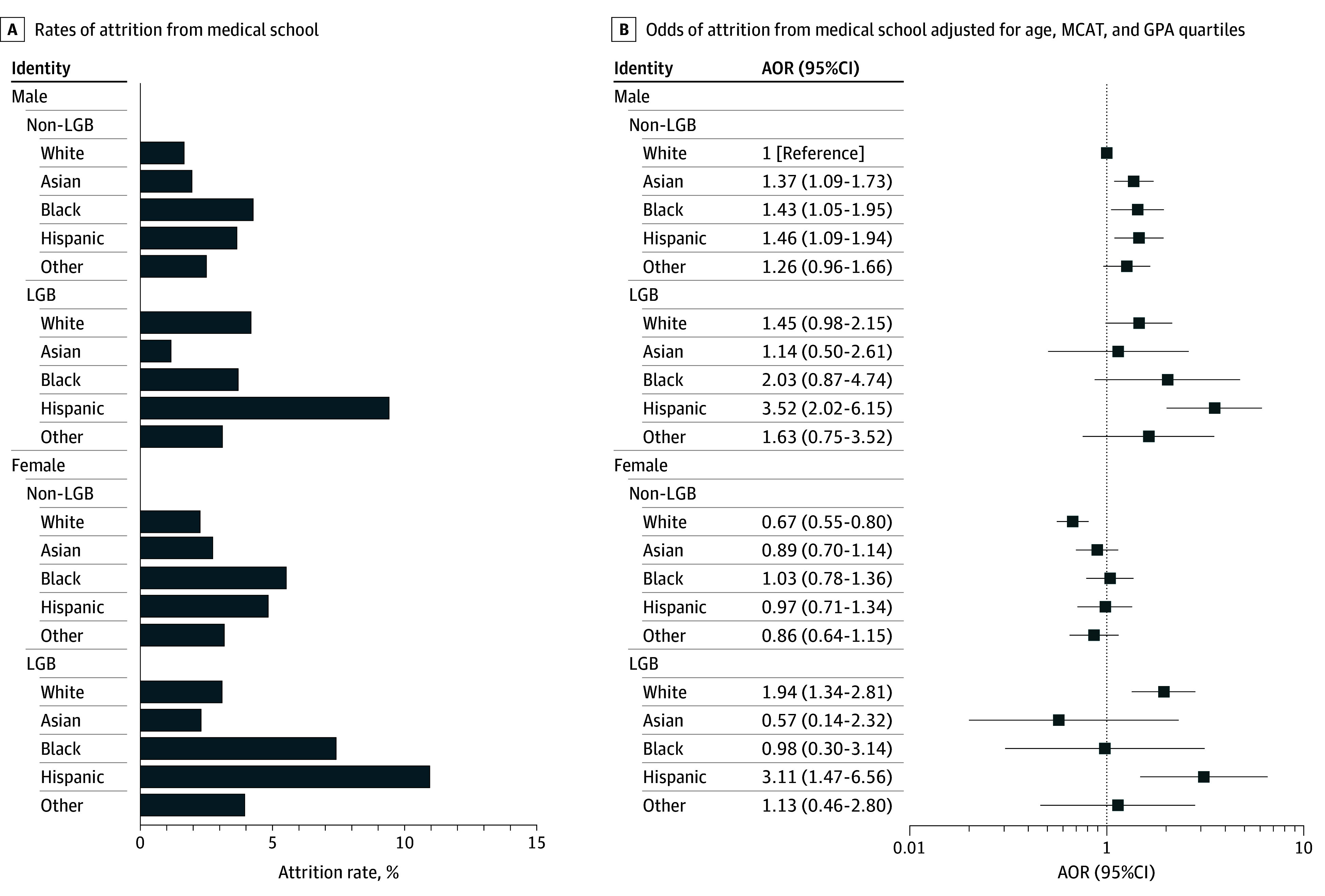
Association of the Intersectionality of Sexual Orientation, Racial and Ethnic Identity, and Sex With Attrition From Medical School AOR indicates adjusted odds ratio; GPA, grade point average; LGB, lesbian, gay, bisexual; MCAT, Medical College Admission Test.

## Discussion

This cross-sectional study revealed high attrition rates among LGB medical students, with the highest observed among LGB Hispanic male and female students. Our findings highlight the importance of intersectionality in understanding attrition from medical school. Although future studies need to examine the cause of these disparities in attrition, LGB students experience discrimination within medical training environments,^2^ which may lead to risk of attrition.^3^

Members of both the LGB and Hispanic communities may encounter less supportive attitudes toward homosexuality,^4^ often more prevalent among recent immigrants or first-generation households, and traditional cultural values around notions of masculinity, authority, and gender roles, perpetuating rigid expectations around sexuality and gender expression and alienating Hispanic LGB students.^5^ Trends in medical education have not explicitly included Hispanic-specific discrimination issues, inadvertently reducing social support for Hispanic students.^6^ Future qualitative studies should explore these intersecting challenges and address the elevated attrition rates.

This study was limited by self-reported data and our inability to examine attrition rates for other marginalized sexual orientations (asexual, pansexual), genders (transgender), and Hispanic nationalities. Higher attrition rates among MSQ nonrespondents suggested that disengagement may be associated with subsequent attrition. Examining medical school attrition rates among marginalized identities and contextualized experiences at the intersection of these identities is essential to developing support services that empower student success.

## Notes

Supplement.
Data Sharing Statement
