# Supplementary material for: Intersectionality of Sexual Orientation, Race, and Ethnicity in Medical School Attrition
Source: JAMA Netw Open. 2025 Jun 10;8(6):e2514515. doi: 10.1001/jamanetworkopen.2025.14515 (PMC12152700; doi:10.1001/jamanetworkopen.2025.14515)
Supplement: Supplement. — Data Sharing Statement [file jamanetwopen-e2514515-s001.pdf]

Data Sharing Statement

Nguyen. Intersectionality of Sexual Orientation, Race, and Ethnicity in Medical School Attrition. *JAMA Netw Open*. Published June 10, 2025. doi:10.1001/jamanetworkopen.2025.14515

Data
Data available: No
